# Efficacy and safety of intravenous laronidase for mucopolysaccharidosis type I: A systematic review and meta-analysis

**DOI:** 10.1371/journal.pone.0184065

**Published:** 2017-08-31

**Authors:** Alícia Dorneles Dornelles, Osvaldo Artigalás, André Anjos da Silva, Dora Lucia Vallejo Ardila, Taciane Alegra, Tiago Veiga Pereira, Filippo Pinto e Vairo, Ida Vanessa Doederlein Schwartz

**Affiliations:** 1 Postgraduate Program in Genetics Applied to Medicine, Department of Pediatrics, Faculdade de Medicina, Universidade Federal do Rio Grande do Sul, Porto Alegre, RS, Brazil; 2 Hospital Materno-Infantil Presidente Vargas, Porto Alegre, RS, Brazil; 3 Clinical Genetics Unit, Children's Hospital, Grupo Hospitalar Conceição, Porto Alegre, RS, Brazil; 4 UNIVATES University, Lajeado, RS, Brazil; 5 Department of Genetics, Universidade Federal do Rio Grande do Sul, Porto Alegre, RS, Brazil; 6 Nutrition, Biomarkers and Health Research Group, University College Dublin, Dublin, Ireland; 7 Instituto de Educação e Ciências em Saúde, Hospital Alemão Osvaldo Cruz, São Paulo, SP, Brazil; 8 Medical Genetics Service, Hospital de Clínicas de Porto Alegre, Porto Alegre, RS, Brazil; 9 BRAIN Laboratory, Hospital de Clínicas de Porto Alegre, Porto Alegre, RS, Brazil; Azienda Ospedaliero-Universitaria Santa Maria della Misericordia, ITALY

## Abstract

Mucopolysaccharidosis type I (MPS I) is an autosomal recessive disease caused by deficient activity of alpha-L-iduronidase. Intravenous (IV) enzyme replacement therapy (ERT) with laronidase is currently used for treating patients with MPS I.

**Objective:**

To evaluate the efficacy and safety of IV laronidase for MPS I.

**Methods:**

A systematic literature review was performed by searching the ClinicalTrials.gov, MEDLINE/PubMed, EMBASE, LILACS, and Cochrane Library databases, limited to clinical trials published until December 31, 2016. The first inclusion criterion was being a randomized controlled trial (RCT). If < five RCTs were identified, open-label and nonrandomized trials, controlled or uncontrolled (quasi-experimental), including ≥ five patients, and evaluating relevant outcomes defined *a priori*, would also be included. For meta-analysis, primary inferences were based on random-effects models. Assessment of article quality was performed in accordance with the GRADE criteria. The Cochrane Risk of Bias tool was used to examine the risk of bias for RCTs.

**Results:**

The selection phase retrieved 632 articles. During the first phase of selection, 158 had the abstract or full text read for assessment of eligibility, of which nine (two RCTs) were included for qualitative synthesis. Four papers were included in the meta-analysis, which was performed for the following outcomes: occurrence of treatment-emergent or infusion-related adverse events (65%; 95%CI 53, 76), mild in most cases; development of IgG antibodies to laronidase (88%; 95%CI 67, 100); apnea-hypopnea index (not significant—NS), urinary glycosaminoglycans (GAGs) [mean change -65.5 μg/mg creatinine (95%CI -68.8, -62.3)], liver size [mean change -31.03% (95%CI -36.1, -25.9)], left ventricular mass index (LVMI) [mean change -1.8 (95%CI -2.32, -0.25)], and distance covered in the 6-minute walk test (NS). Among the outcomes not included in meta-analysis, we found evidence for benefit of laronidase only on shoulder flexion.

**Conclusions:**

Our findings suggest that IV laronidase effectively reduces urinary GAGs excretion, hepatomegaly and LVMI, and can improve shoulder flexion in MPS I patients. Laronidase appears to be safe in the studied population.

## Introduction

Mucopolysaccharidosis type I (MPS I; OMIM 252800) is an autosomal recessive lysosomal storage disease (LSD) caused by deficient activity of alpha-L-iduronidase (IDUA, EC 3.2.1.76). IDUA catalyzes cleavage of iduronic acid residues from the glycosaminoglycans (GAGs) heparan and dermatan sulfate. In MPS I, these partially broken down GAGs build up within lysosomes and are excreted in urine at above-normal concentrations [1].

The worldwide incidence of this disease is estimated at approximately 1 in 100,000 newborns [1–3]. However, neonatal screening data have yielded incidence rates ranging from 1:7,353 (Washington state, USA) [4] to 1:14,567 (Missouri, USA) [5].

Patients are affected to different degrees, but life expectancy is generally shorter than the healthy individuals [1]. From a clinical standpoint, cases can be divided into three broad phenotypes: Hurler syndrome, the severe form, with major intellectual impairment; Hurler–Scheie syndrome, the intermediate form, and Scheie syndrome, the attenuated form, the latter with little or no cognitive impairment. However, it bears noting that these subtypes are part of a continuous spectrum representing a single condition, and that clinical heterogeneity is observed even within subgroups [3].

There is no curative treatment for MPS I. Currently available therapeutic options include interventions designed to address the clinical phenotype (such as surgery to correct hernias) or the mutant protein (hematopoietic stem-cell transplantation and enzyme replacement therapy [ERT] with laronidase). Laronidase, or recombinant α-L-iduronidase (trade name: Aldurazyme), is manufactured by Biomarin Pharmaceutical Inc. and Genzyme Corporation, USA. This enzyme is produced by recombinant DNA technology in Chinese hamster ovary cells. The recommended dosage regimen of laronidase is 0.58 mg/kg body weight, administered once weekly as an intravenous (IV) infusion. Pretreatment is recommended 60 minutes prior to the start of the infusion and may include antihistamines, antipyretics, or both [6]. At the currently recommended dosage, laronidase does not cross the blood–brain barrier and, therefore, has no effect on the central nervous system. Three published systematic reviews have aimed to assess laronidase efficacy and safety [7–9]. Two of these [8, 9] included only one phase III randomized clinical trial (RCT) [10]. One of these systematic reviews [7] also included a follow-up study [11], two case reports [12, 13], and one uncontrolled clinical trial [14]. None of these reviews included a meta-analysis. Therefore, the impact of laronidase treatment on key outcomes is still unclear. Doubts remain, for instance, as to the ideal timing of ERT initiation and whether ERT has similar efficacy in the Hurler, Hurler–Scheie, and Scheie forms.

The present study sought to evaluate the efficacy and safety of laronidase ERT in MPS I by conducting a systematic review with meta-analysis with expanded study inclusion criteria, i.e., considering non-RCT designs eligible for meta-analysis.

## Methods

This study has been registered with the PROSPERO database (CRD42015032570). The systematic review was conducted as proposed by the Cochrane Collaboration [15] and reported as proposed by PRISMA Statement (S1 Table).

### Information sources and search strategy

The MEDLINE (via PubMed), Embase, LILACS, and Cochrane Central Register of Controlled Trials databases were searched. The ClinicalTrials.gov website was also searched for potentially eligible trials. The only restriction on this search strategy was the date of publication. Only those studies published before December 31, 2016 were retrieved. The following search strategy was applied to MEDLINE/PubMed: (MUCOPOLYSACCHARIDOSIS TYPE I OR MUCOPOLYSACCHARIDOSIS I) AND (LARONIDASE OR ENZYME REPLACEMENT THERAPY). For Embase and LILACS, an expanded search query with the following keywords was used: mucopolysaccharidosis AND type AND i OR 'mucopolysaccharidosis'/exp OR mucopolysaccharidosis AND i combined with 'laronidase'/exp OR laronidase OR 'enzyme'/exp OR enzyme AND replacement AND ('therapy'/exp OR therapy).

### Eligibility criteria and study selection

We planned to include RCTs of laronidase ERT for treatment of patients with a confirmed diagnosis of MPS I. If < five trials meeting these criteria were identified, lower-powered studies (open-label and non-randomized trials, controlled or otherwise, including quasi-experimental designs), as long as the sample size was ≥ five, would also be included. Studies that did not evaluate at least one of the 28 outcomes of interest defined *a priori* by a team of experts, were excluded (Table 1).

**Table 1:** Laronidase for MPS I: Outcomes defined *a priori* and studies that evaluated them.

Outcomes	Number of articles	References included in meta-analysis	References excluded from meta-analysis	Reasons for exclusion
**Adverse events**	6	[11, 14, 16, 17]	[10, 18]	same population
**AHI**	6	[11, 19]	[10, 18]	same population
			[14, 16]	no MD
**GAGs**	6	[11, 14, 16, 17]	[10, 18]	same population
**Hepatomegaly**	6	[11, 16, 17]	[10, 18]	same population
			[14]	different measurement method
**Cardiomyopathy**	5	[14, 20]	[18]	same population
			[16]	different measurement method
			[11]	data not shown
**Joint mobility**	5		[10, 18]	same population
			[16]	no MD
			[11]	only study available
			[21]	unclear measurement method
**Height and growth velocity**	4		[16]	only study available
			[11, 14]	data not shown
			[18]	same population
**Quality of life**	4		[10]	same population
			[11]	only study available
			[21]	no MD
			[18]	different measurement method
**Valvulopathy**	4		[11, 16, 20]	data not shown
			[18]	same population
**Corneal clouding**	3		[16, 18]	data not shown
			[18]	same population
**Death**	3	[11, 14, 17]		
**6-minute walk test performance**	3	[11, 17]	[10]	same population
**Visual acuity**	3		[16]	data not shown
			[18]	same population
			[11]	only study available
**Weight**	3		[14, 16]	data not shown
			[18]	same population
**Functional classification (NYHA)**	2		[16]	only study available
			[18]	same population
**FVC**	2		[10]	same population
			[11]	only study available
**Splenomegaly**	2		[16]	only study available
			[18]	same population
**Myelopathy and hydrocephalus**	1		[18]	only study available
**Neurological regression**	1		[14]	only study available
**Nutritional status**	0			
**Number of RTIs**	0			
**Number of hospitalizations**	0			
**Number of ear infections**	0			
**Intellectual disability**	0			
**Survival**	0			
**Cost-effectiveness**	0			
**Behavioral disorders**	0			
**12-minute walk test performance**	0			

AHI = apnea-hypopnea index; FVC = forced vital capacity; RTI = respiratory tract infection; NYHA = New York Heart Association; MD = measure of dispersion.

The selection stage was performed independently by two investigators (ADD, DLVA), who assessed the abstracts and full text retrieved during the search stage. Decisions were compared, and articles deemed relevant were forwarded to two other investigators (OA, AAS) who, independently, using standardized data collection forms, extracted information on the characteristics of these studies (design, randomization methods, population, interventions, and outcomes). The two investigators then took part in a consensus meeting. Any divergences that remained were addressed by the intervention of a third investigator (IVDS). Finally, the references of the selected articles were hand searched for potentially relevant studies not identified by the previous search strategies. During the data extraction process, the ClinicalTrials.gov database and Cochrane Central Register of Controlled trials were searched for supplemental information not present in the articles. When such information could not be retrieved, an email was sent to authors requesting non-reported data.

Studies with overlapping data were excluded from meta-analyses. In these cases, the study with the largest sample (or, if both studies had the same sample size, that with the longest follow-up) was retained for analysis. The Cochrane Risk of Bias (RoB) tool was used to examine the risk of bias in RCTs. Each domain was classified as having "low risk" (+), "high risk" (-) or unclear risk (?) of bias.

### Statistical analysis

For meta-analysis, we used both fixed and random-effects (RE) models based on the general inverse variance approach and on the DerSimonian-Laird method, respectively. Primary inferences are based on RE analyses. By assuming a normal distribution, we calculated approximate standard error estimates from 95% confidence intervals (CI). For continuous outcome data (e.g. mean change from baseline), when not reported, missing standard deviations (SD) were imputed using the sample size weighted SD from all of the studies with complete information. Meta-analyses of proportions employed the Freeman-Tukey double arcsine transformation. For single studies involving count data, exact 95% CI were calculated. We also computed 95% predictive intervals, that is, the approximate predictive distribution for an effect in a future study, given the current observed levels of statistical heterogeneity and uncertainty. For the latter analyses, we used the frequentist approach described by Higgins et al. [22]. We detected and quantified the statistical heterogeneity via the Cochran's Q test and I^2^ metric, respectively. Results with P < 0.05 were deemed statistically significant, except for the Q test, which was considered significant when a P <0.10 was observed [23]. Within the same trial, when multiple groups were investigated (e.g. different doses were tested), each group was considered an independent stratum. Depending on the number of studies found, we planned to do funnel plots and tests for small study bias and publication bias. All analyses were carried out with Stata 13 (StataCorp, College Station, TX, USA).

### Analysis of outcomes not included in meta-analysis

We defined *a priori* that only those outcomes reported in > 50% of studies included in the systematic review (e.g, in at least four papers) would be compared and selected for systematic evaluation of quality according to the GRADE criteria [24–26]. Furthermore, only those outcomes compared before and after intervention and with a GRADE classification of moderate or better were considered indicative of evidence of efficacy or safety. Assessment of article quality in accordance with the GRADE criteria was performed independently by two investigators (ADD, TA).

## Results

**Fig 1:**
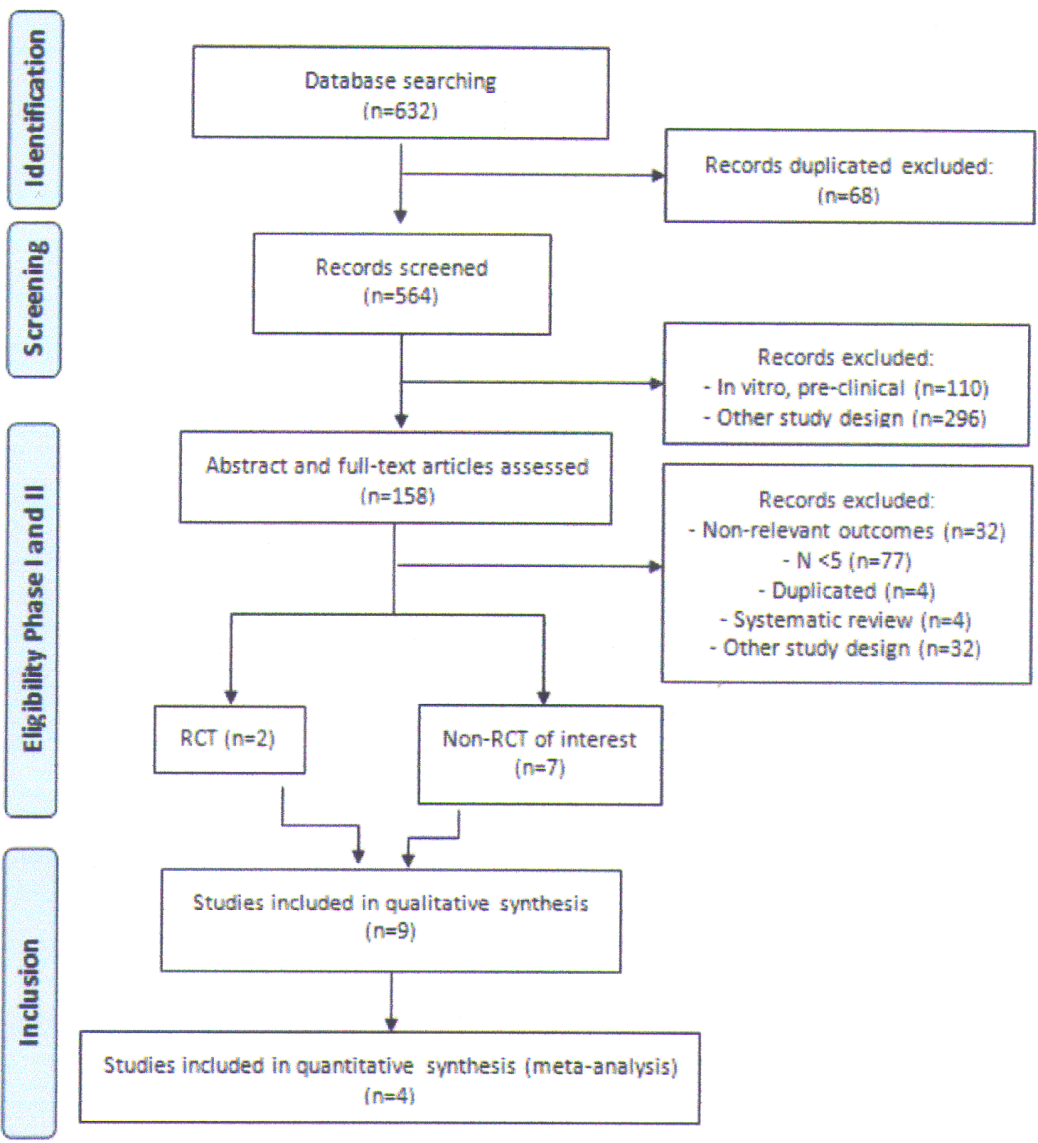
Laronidase for MPS I: PRISMA flow diagram.

The search results are described in Fig 1. The broad search strategy retrieved 632 articles. During the first phase of selection, 68 duplicated entries were excluded and 564 articles were screened, of which 158 were read in full for assessment of eligibility. Out of these articles, four were systematic reviews [7–9, 27] that included data from other included articles and were thus excluded from selection. As only two papers were RCTs, we expanded our selection criteria as defined *a priori*. Then, we conducted a second round of screening; this yielded nine papers, including the two RCTs (Table 2). As defined *a priori*, two studies reporting on data from the same population were excluded from data extraction [10, 18].

**Table 2:** Systematic review of IV laronidase for MPS I: Characteristics of included studies (n = 9).

Author	N	Design	Laronidase IV	Premedication	Study duration (weeks)	Age at baseline (years; range)	Severity of phenotype (n)	Primary outcomes
**Kakkis et al. [16]**	10	Quasi-experiment	0.58 mg/kg weekly	Diphenhydramine as needed	52	12.4 (mean; 5–22)	H = 1; HS = 8; S = 1	Organomegaly and GAGs
**Wraith et al. [10]**	22 vs 23	DB, multicenter RCT	0.58 mg/kg weekly vs. placebo	As needed^#^	26	15.5 (mean; 6–43)	H = 1; HS = 37; S = 7	FVC and 6MWT
**Wraith et al. [14]**	16 vs 4	OL multicenter controlled trial	0.58 mg/kg weekly vs. 1.16 mg/kg weekly	As needed^#^	52	2.9 (mean; 0.5–5.1)	H = 16; HS = 4	Safety
Sifuentes et al. [18]*	5	Extension of Kakkis et al. [16]	0.58 mg/kg weekly	Not described	288	12 (mean; 9–17)	HS = 4; S = 1	Organomegaly and GAGs
Clarke et al. [11]*	45	Multicenter, uncontrolled extension trial	0.58 mg/kg weekly	As needed^#^	182 or 208	15.7 (mean; 6.3–43.3)	HS = 38; S = 7	FVC and 6MWT
**Giugliani et al. [17]**	8 vs 8 vs 8 vs 9	OL multicenter RCT	0.58 mg/kg weekly vs. 1.2 mg/kg every 2 weeks vs. 1.2 mg/kg weekly vs. 1.8 mg/kg every 2 weeks	As needed^#^	26 or 27	8.9 (mean; 1.4–20.7)	H = 10; HS = 16; S = 7	GAGs
**Tylki-Szymanska et al. [21]**	17	Quasi-experiment	0.58 mg/kg weekly	Not described	208	8.1 (mean; 1–39)	H = 10; HS = 2; S = 5	ROM and functional status
**Brands et al. [20]**	8	Quasi-experiment	0.58 mg/kg weekly	Not described	193.6	5 (mean; 1.3–9.3)	H = 5; S = 3	Cardiovascular abnormalities
**Dualibi et al. [19]**	9	Quasi-experiment	0.54 mg/kg weekly	Not described	88	8 (median; 3–20)	HS = 8; S = 1	Sleep and hearing disorders
Total*	142	-	-	-	Range: 26–208	Range: 0.5–43.3	H = 43; HS = 75; S = 24	

*Sifuentes et al. **[18]** is a follow up study of Kakkis et al. **[16].** Clarke et al. **[11]** is a follow up study of Wraith et al. **[10]**. Data from same study not included in total.

^#^Antihistamines or antipyretics were prescribed as needed. IV = intravenous; OL = open-label; RCT = randomized clinical trial; DB = double-blind; GAGs = urinary glycosaminoglycans; FVC = forced vital capacity; 6MWT = 6-minute walk test; ROM = range of motion.

In the nine articles included, data were available for 19 of the 28 outcomes of interest. Among the four papers with data available for meta-analysis, only seven of 28 outcomes (adverse events, apnea-hypopnea index—AHI, GAGs, liver volume, cardiomyopathy, and performance in 6-minute walk test performance) could be assessed by meta-analysis (Table 1). Reasons for exclusion of studies from meta-analysis are also listed in Table 1. Qualitative synthesis could be performed for four of 21 other outcomes: joint mobility, height, quality of life, and heart valve involvement.

Publication bias was present in included studies. Funnel plots and tests for small study bias and publication bias could not be carried out because of the limited number of studies available (e.g. n < 10 studies) [28]. Due to the absence of suitable comparator groups and the small number of studies included in the meta-analyzes, it was not possible to estimate inflation in relative efficiency magnitudes, nor to employ funnel plot asymmetry tests.

Regarding co-interventions (Table 2), in Kakkis et al. [16] diphenhydramine was used at the physician's discretion as premedication in patients with a history of urticaria. Kakkis et al. [16], Sifuentes et al. [18], Wraith et al. [10], Clarke et al. [11] and Giugliani et al. [17] used human albumin to dilute laronidase. Only Clarke et al. [11] referred no difference in safety profile among patients who received laronidase without human albumin administration. Wraith et al. [14] did not use albumin, and reported a similar adverse event profile. In Kakkis et al. [16], when clinically indicated, patients underwent cervical fixation for subluxation, mitral valve replacement, and ventriculo-peritoneal shunt placement (each procedure in a different patient) during the trial. Dualibi et al. [19] reported the following procedures in their sample: one patient underwent myringotomy with tube placement and adenoidectomy, another patient underwent adenotonsillectomy, and two other patients underwent tympanostomy with tube placement. All surgeries happened prior to ERT starting; however, the time between surgery and AHI evaluation is not clear. The other studies included in the review did not report any co-interventions other than those already described.

### Data included in meta-analysis

**Fig 2:**
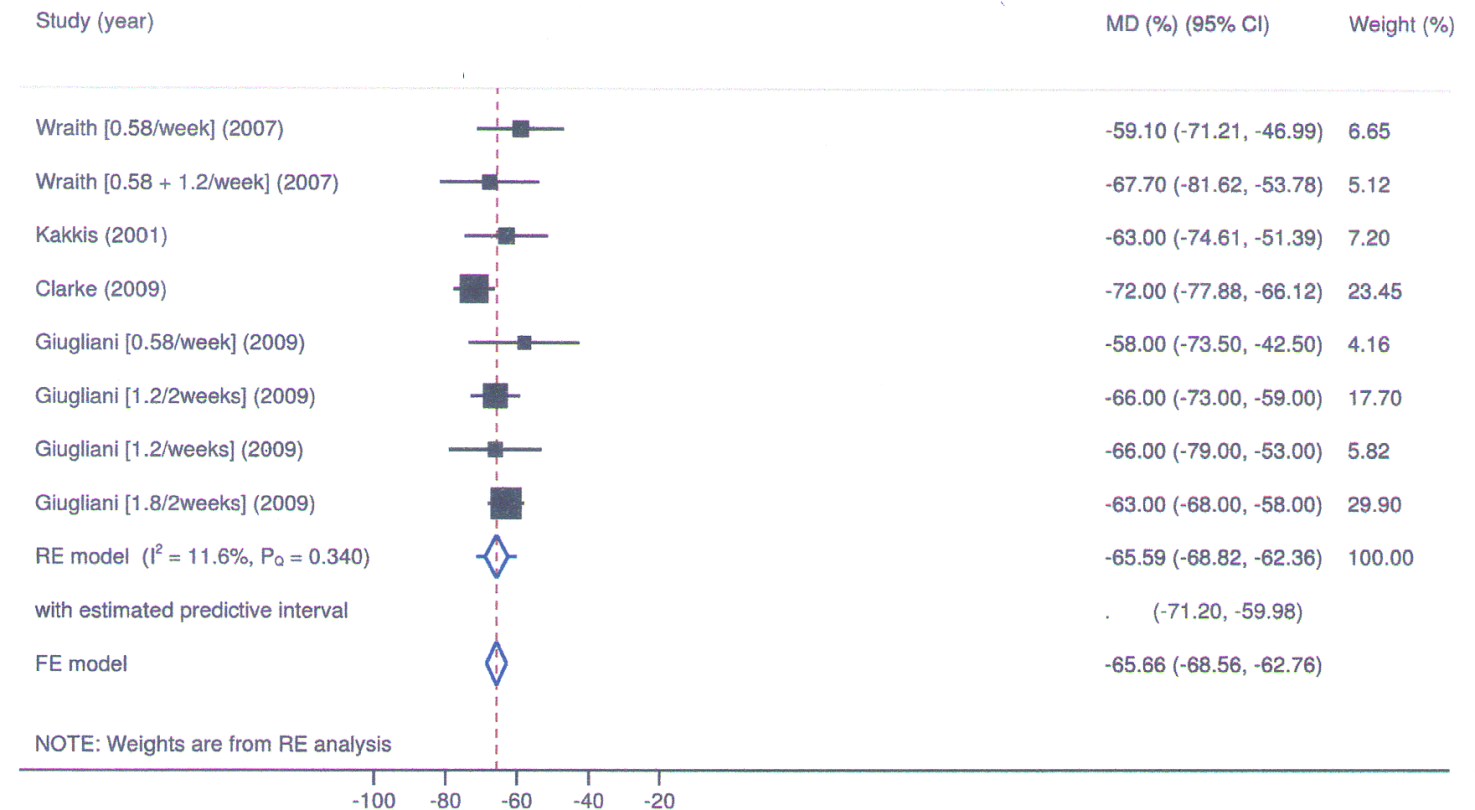
Laronidase for MPS I: Forest plot for mean change in urinary GAGs excretion under fixed and random-effects models.

**Fig 3:**
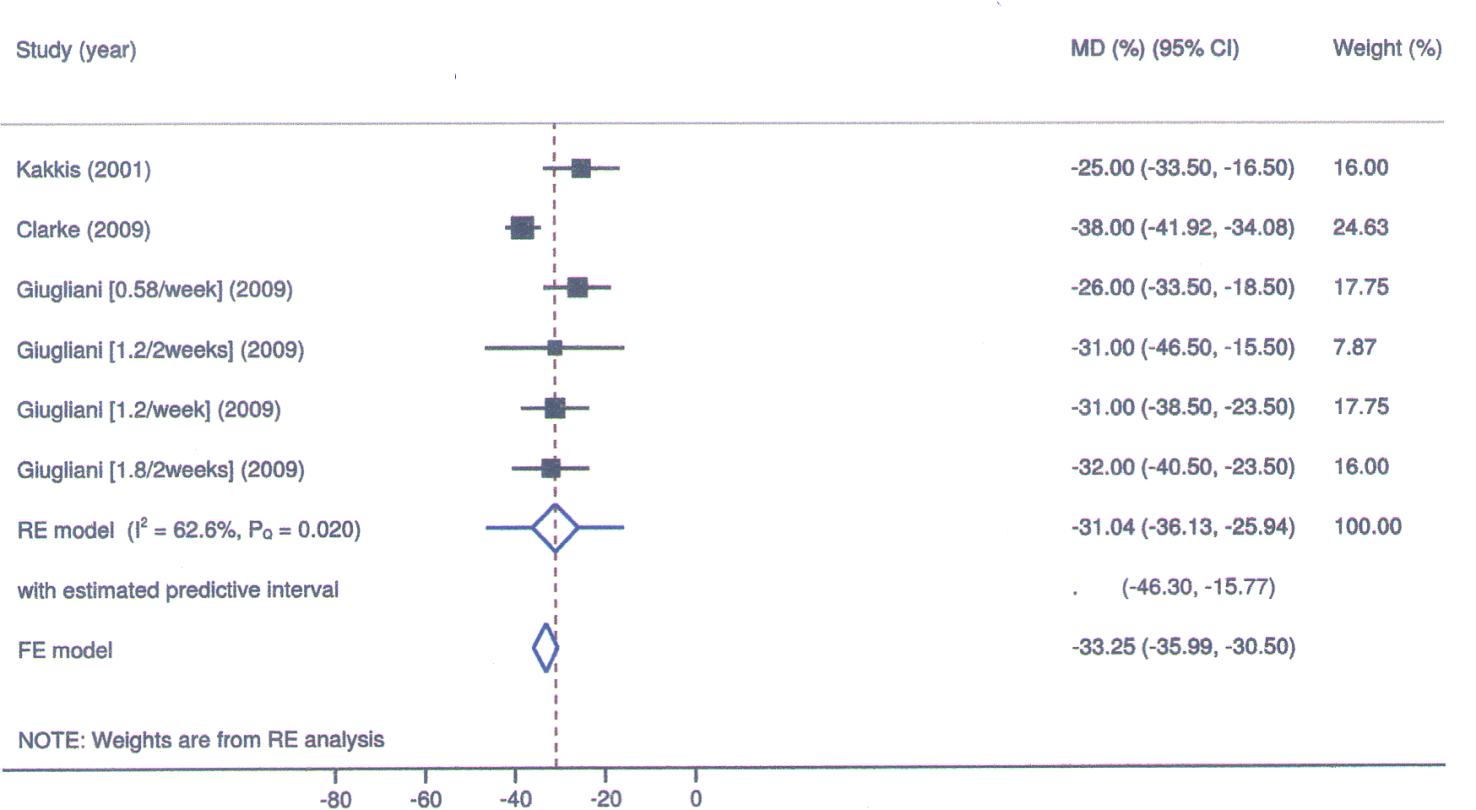
Laronidase for MPS I: Forest plot for mean change in liver volume under fixed and random-effects models.

**Fig 4:**
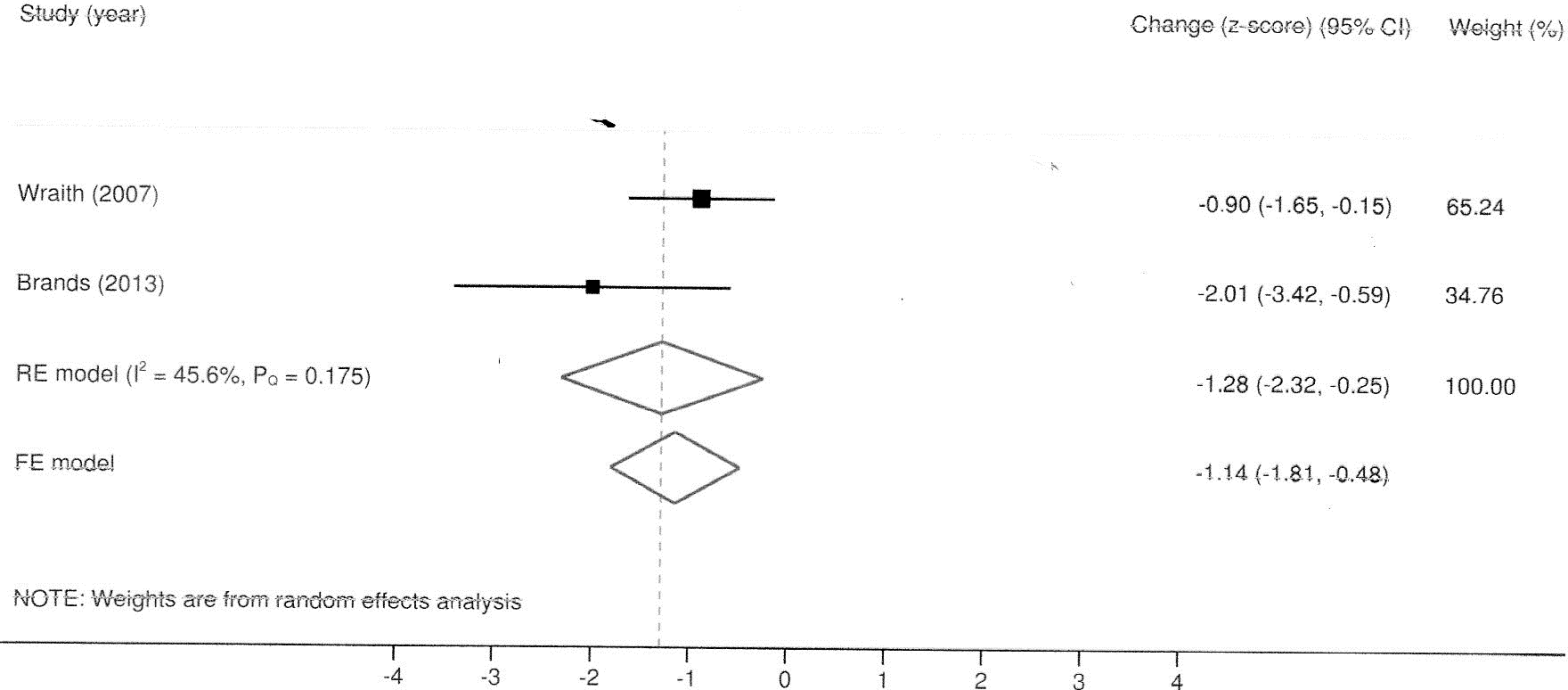
Laronidase for MPS I: Forest plot for mean change in left ventricular mass index under fixed and random-effects models.

**Fig 5:**
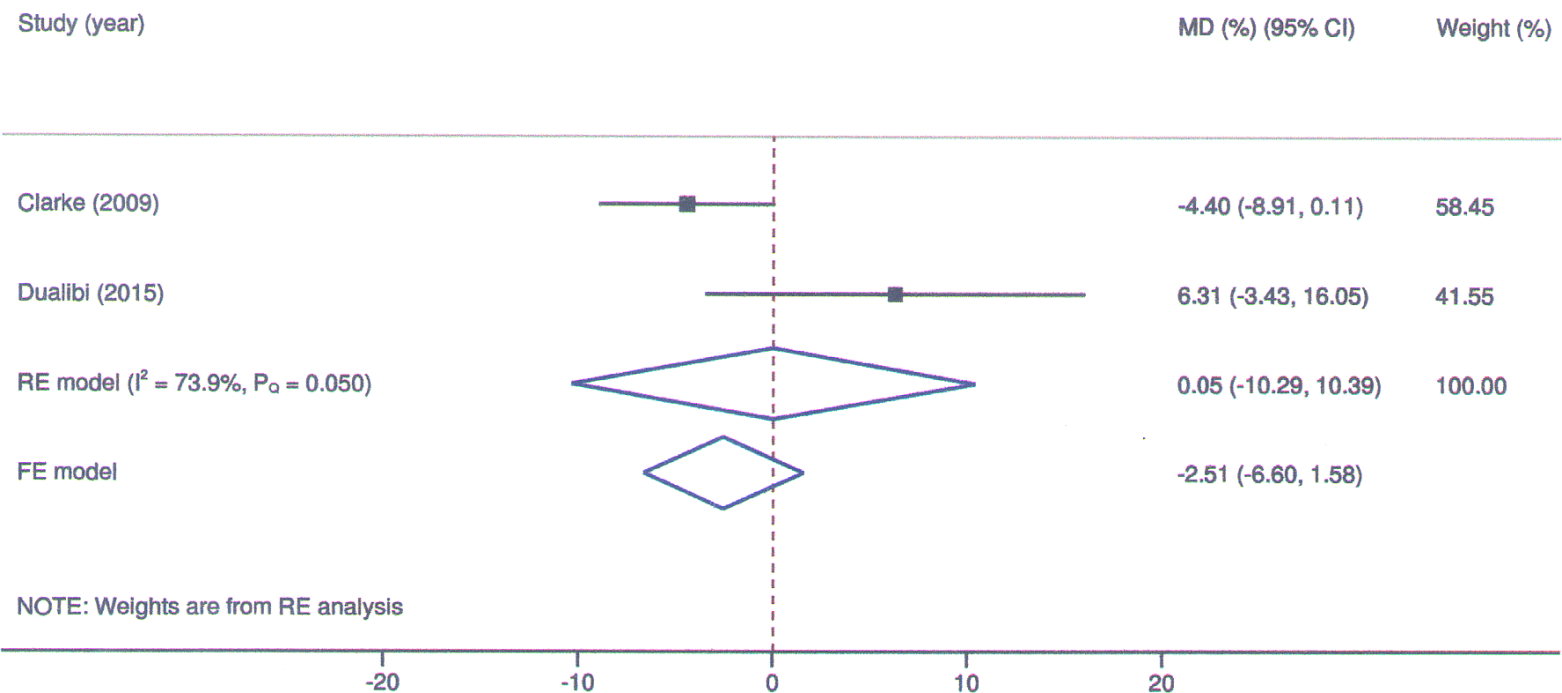
Laronidase for MPS I: Forest plot for mean change in apnea-hypopnea index under fixed and random-effects models.

**Fig 6:**
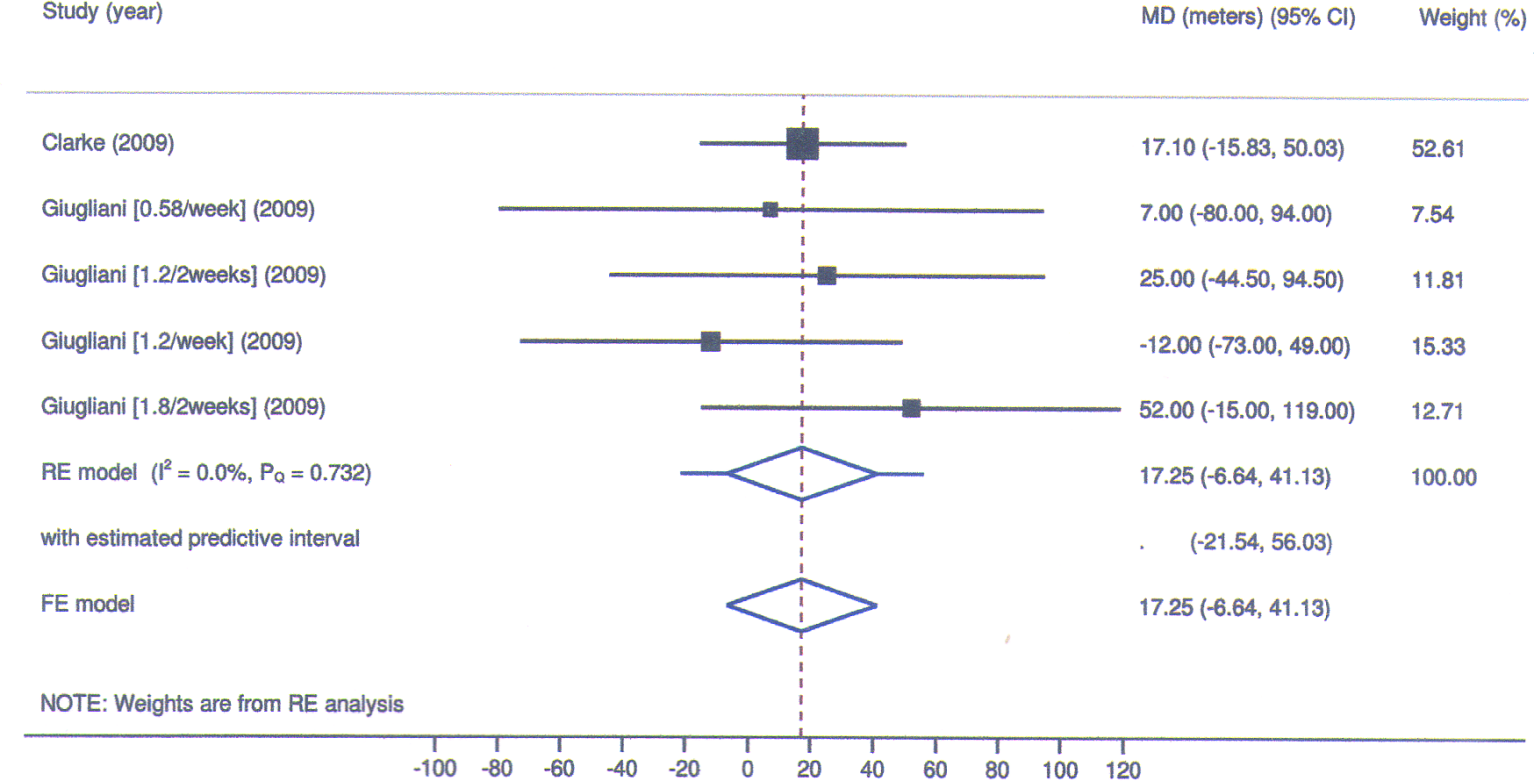
Laronidase for MPS I: Forest plot for mean change in 6-minute walk test performance under fixed and random-effects models.

The results of meta-analysis for seroconversion and frequency of adverse events, mean change in AHI, GAGs, liver volume, cardiomyopathy, and performance in 6-minute walk test (6MWT) are described in Figs 2–6 and in Tables 3 and 4. All reported deaths (n = 4) were deemed unrelated to treatment. We observed missing standard deviations/standard errors for the study by Kakkis et al. only. The following outcomes had imputed SD and will be further discussed: GAGs and liver volume. The evaluation of the risk of bias in RCTs included is shown in Table 5.

**Table 3:** Laronidase for MPS I: Laboratory markers of safety (latest follow-up vs baseline).

Adverse event	Number of studies*	N	Summary results		P-value for Cochran's Q test	I^2^	References
			Fixed Effect	Random Effect		(95% CI)	
			(95% CI)	(95% CI)			
Urticaria	5	43	18%	17%	0.110	47%	[16, 17]
			(7–32%)	(3–37%)		(0–81%)	
Rash	5	78	17%	16%	0.297	18.3%	[11, 17]
			(8–27%)	(6–28%)		(0–83%)	
Fever	7	108	16%	17%	0.275	20.2%	[11, 14, 16, 17]
			(9–25%)	(8–27%)		(0–64%)	
Drug-related AEs	5	78	65%	65%	0.625	0%	[11, 17]
			(53–76%)	(53–76%)		(0–79%)	
Any IRR	6	98	45%	44%	0.399	2.7%	[11, 14, 17]
			(34–55%)	(34–55%)		(0–75%)	
Death	6	98	2%	2%	0.660	0%	[11, 14, 17]
			(0–7%)	(0–7%)		(0–75%)	

*In the Giugliani et al. study, each group was considered an independent stratum.

**Table 4:** Treatment-emergent adverse events during laronidase therapy for MPS I.

Outcome	Number of studies*	N	Summary results		P-value for Cochran's Q test	I^2^ (95% CI)	References
			Fixed Effect (95% CI)	Random Effect (95% CI)			
Seroconversion to laronidase	6	98	91%	88%	0.0006	82.6%	[11, 14, 17]
			(85–96%)	(67–100%)		(55–93%)	
Platelet count decreased	6	63	4%	5%	0.081	49%	[14, 16, 17]
			(0–12%)	(0–18%)		(0–80%)	
Platelet count increased	6	63	1%	1%	0.553	0%	[14, 16, 17]
			(0–7%)	(0–7%)		(0–75%)	
White blood cell disorder	6	63	1%	1%	0.553	0%	[14, 16, 17]
			(0–7%)	(0–7%)		(0–75%)	

*In the Giugliani et al. study, each group was considered an independent stratum.

AE = adverse event; IRR = infusion-related reaction.

**Table 5:** Risk of bias judgments for the included randomized trials.

Study	Random sequence generation (selection bias)	Allocation concealment (selection bias)	Blinding of participants and personnel (performance bias)	Blinding of outcome assessment (detection bias)	Incomplete outcome data (attrition bias)	Selective outcome reporting (reporting bias)	Other bias
**Giugliani et al. [17]**	?	?	-	+	+	+	-
**Wraith et al. [10]**	?	?	?	+	+	+	-

+ = low risk of bias;— = high risk of bias;? = unclear risk of bias.

In the studies of Wraith et al. [14] and Clarke et al [11], GAGs levels in urine presented a mean reduction of 61.3% and 72%, respectively. In Giugliani et al [17], the four treatment regimens were effective in reducing urinary GAGs levels, with a 58% reduction at a dose of 0.58mg/kg/week. In the study by Kakkis et al [16], there was a 63% reduction in GAGs. In Clarke et al. [11] and in Giugliani et al. [17] the hepatic volume decreased by 38% and 26%, respectively, being this reduction described in the group 0.58mg/kg/week. In the study by Kakkis et al [16], there was a 25% decrease in hepatic volume.

### Qualitative synthesis of non-meta-analyzed data

Qualitative synthesis was performed for the following outcomes not included in meta-analysis: joint mobility, quality of life (QoL), valve involvement, and height (Table 6). Evidence of benefit of laronidase therapy was only found for joint mobility. For the other three outcomes, the data available in the literature was insufficient for us to draw any inferences as to the effect of the drug (GRADE classification low to very low, Table 6).

**Table 6:** Laronidase for MPS I: Relevant outcomes not included in meta-analysis.

	Joint mobility (active ROM)	Height and growth velocity	Quality of life	Valvulopathy
**Kakkis et al. [16]**	RSF ↑ 28° (p<0.001); RSF ↑ 26° (p<0.002); REE ↑ 7° (p = 0.03); REE ↑ 7° (p = 0.007)	Growth rate ↑ 85% (p = 0.01)	NE	NA
**Wraith et al. [10]**	NA	NE	NA	NE
**Wraith et al. [14]**	NE	NA	NE	NE
**Sifuentes et al. [18]**	NA	NA	NA	NA
**Clarke et al. [11]**	SF ↑ 17.4° (p<0.001)	NA	NA	NA
**Tylki-Szymanska et al. [21]**	SF ↑ 33.5° (p = 0.046); EE ↑ 4.3° (p = 0.225)	NE	HAQ ↑ (p<0.05)	NE
**Brands et al. [20]**	NE	NE	NE	NA
**Dualibi et al. [19]**	NE	NE	NE	NE
**GRADE**	MODERATE	LOW	LOW	VERY LOW

Data are presented for outcomes mentioned in four or more studies and from studies that included statistical analysis of change from baseline. Data on adverse events are presented separately. NA = p-value not available; NE = not evaluated; ROM = range of motion; RSF and LSF = right and left shoulder flexion (SF) respectively; REE and LEE = right and left elbow extension (EE) respectively; HAQ = Health Assessment Questionnaire.

#### Joint mobility (GRADE moderate)

In all included articles that reported this outcome (n = 5), joint mobility was evaluated by range of motion (ROM), measured by a goniometer (Table 1). However, the studies were heterogeneous, as the protocols used for ROM evaluation differ in regards to the side measured, the type of ROM (active or passive), and the joints evaluated. GRADE classification for this outcome was MODERATE (Table 6), suggesting that laronidase ERT has a beneficial effect on shoulder flexion (improvement in ROM ranging from 17° to 33.5°).

#### Height and growth velocity (GRADE low)

Height was evaluated in four studies (Table 1). However, only one reported statistical analyses (Table 6). Kakkis et al. [16] reported a mean 6 cm (5%) increase in height in six prepubertal patients; mean growth velocity also increased (Table 6). Sifuentes et al. [18] reported greater gain of height in the group that started receiving ERT at the prepubertal stage, with a mean gain of 33 cm after 6 years, representing 27% growth from baseline.

#### Quality of life (GRADE low)

The included studies (n = 4) assessed QoL through different instruments: Sifuentes et al. used a modified version of an MPS-specific questionnaire [18], without mentioning details about it. In this study patients answered 100 questions about work, participation in sports and leisure activities. Most patients responded that the main benefits associated with treatment were in energy and endurance, making them relatively independent (personal hygiene, dressing); in quality of sleep; in self-esteem; and in participation in daily activities. Patients reported that they started to have new goals in life, including going to college, having their own home, getting married and having a family, and having fun.

The other studies used the Health Assessment Questionnaire (HAQ), Child HAQ (CHAQ) or MPS-HAQ, which scores range from 0 to 3. In the study by Wraith et al. [10], the CHAQ/HAQ score was 1.9 in the placebo group and 2.0 in the laronidase group at first assessment, with no significant between-group difference after treatment. Tylki-Szymanska et al. [21] reported improvement in the following categories of MPS-HAQ (Table 6): eating/drinking (p = 0.028), dressing (p = 0.046), tooth brushing (p = 0.043), toileting (p = 0.028), and walking (p = 0.028).

#### Valvulopathy (GRADE very low)

Valvulopathy was evaluated trough echocardiogram in four studies (Table 1) and no study has shown statistical analysis. At Brands et al. [20], all patients have valve abnormalities at the beginning of treatment (the main findings are mitral valve regurgitation in three patients, aortic valve regurgitation in two and regurgitation of both mitral and aortic valve in one patient).

## Discussion

This is the first meta-analysis to evaluate the safety and efficacy of IV laronidase for treatment of MPS I patients from all age groups, and the first review to systematically include data from all age groups from non-RCT studies for this disorder. Our meta-analysis results show that laronidase effectively reduces liver volume (one of the main markers of GAGs storage), urinary GAGs excretion and LVMI. The effect of IV laronidase in reducing hepatomegaly had previously been reported in a retrospective controlled trial carried out by our group [29].

The results of our systematic review suggest that, as expected for rare disorders, there are few studies on this issue published in the literature. This can be a major limitation of the present meta-analysis, once it precludes the use of graphs and tests for the assessment of funnel plot asymmetry. Therefore, a reduced number of studies is associated with low power to detect underlying statistical heterogeneity [30], so that for outcomes such as GAGs and 6MWT, we cannot rule out the hypothesis that heterogeneity is present but remains statistically undetected [31].

With the advancement of technology, new treatment options are emerging, including those targeting rare diseases. However, without evidence to support or quantify the benefits of these therapies, these options are unlikely to gain widespread use and be covered by health systems. In this case, systematic reviews on costly existing treatments for rare diseases are important to support the clinical decision-making process and to define the profile of patients who are more likely to respond positively to each treatment. When assessing rare diseases, evidence from RCTs is not always available. Therefore, other designs may be considered and assessed critically with a view to supporting the decision-making process.

Other challenge to deal with is the incompletely reported data as publication bias was present, which precludes us from applying GRADEpro® tool, as recommended by Cochrane Collaboration [15]. Usually, data from secondary outcomes were poorly mentioned, once the trial was not designed for measuring that. In most cases, however, there are conclusions being made without showing the data and full report of results.

Four systematic reviews already published and retrieved by our search strategy aimed at evaluating laronidase efficacy and safety. Jameson et al. [9] and El Dib et al. [8] proposed to conduct a meta-analysis; however, the scarcity of data and the limited inclusion criteria precluded this. Jameson et al. [9] included only one study that fulfilled their inclusion criteria, and the same authors were responsible for selection and extraction of data. Noh et al. [7] included two case reports from the same patient, as well as one retrospective study, and did not perform any comparison between the included studies. The only comparison made was between two studies [10, 11], which reported data from the same population.

The fourth systematic review [27], as a component of the safety assessment of laronidase, conducted a meta-analysis, evaluating the presence of anti-laronidase antibodies and shows that although a large number of patients exhibited serum conversion, the results were highly heterogeneous. In addition, an association between the presence of antibodies and the development of adverse effects or the reduction of the treatment effect on the clinical outcomes evaluated (FVC and 6MWT) was not demonstrated in this meta-analysis. This study also shows that patients with greater exposure to anti-laronidase antibodies over time showed greater inhibition of enzyme uptake into cells and may result in a decrease in the pharmacodynamic effect of the exogenously administered therapeutic enzyme.

One strength of the present study was that we included only prospective trials, in an attempt to avoid memory and selection bias. Prospective trials also have the advantage of collecting data accordingly with the aimed outcomes, what doesn’t happen on retrospective trials. Furthermore, including only those case series with n ≥ five increases the statistical power of the findings of our meta-analysis, given that MPS I is a rare disease.

However, a very recently published systematic review and meta-analysis,aiming at the evaluation of Laronidase in adult patients, and that has not been included in the present study due to date of publication [32], included retrospective studies. Their results are in accordance to present study, supporting that laronidase reduces urine GAGs levels and liver volume, while increases the blood anti-ERT antibody levels. The study also suggests, not in accordance with our findings, that laronidase increases the performance on the 6MWT. Methodologically, results previously reported [32] not only considered studies with results showing positive effect towards significance but also included in the analysis results from the same population more than once, and evaluated only adult patients and should be interpreted carefully.

We chose to include studies testing different IV laronidase regimens, as comparisons of these regimens have failed to show significant differences between them [17, 33]. Even if higher doses influence the effect in favor of treatment (not demonstrated in previous studies), we do not believe this would have altered the results of our meta-analysis. The same applies to the occurrence of adverse events at higher doses: current studies do not support statistically significant differences, and this is unlikely to have interfered with our results. [14, 17]

Unpublished work was covered by the identification of conference abstracts containing data deemed to be of interest. The final published articles were then included when available. Brands et al. [34] was a conference-presented abstract of another included article [20] and was thus excluded. Polgreen et al. [35] and Furlan et al. [36] are also abstracts and the final publication of which was not found by our systematic review. The first was excluded as data are not shown separately for each MPS type and the second because the design of the study was not clear.

Although we were not able to perform quantitative measurement of all summarized results, our study also suggests that ERT can affect ROM, with positive effects on shoulder flexion. Interventions such as physical therapy, when provided, may contribute to improved joint mobility, and it is thus advisable that such interventions be reported in all studies that analyze this outcome [37]. However, none of included studies has mentioned this aspect and this lack of information have been reported elsewhere [37].

In the studies included in our review, laronidase was safe, with adverse events being mild in most cases; those reported in two or more studies (and thus included in the meta-analysis) were rash, urticaria, and fever. The only placebo-controlled trial [10] found no significant between-group difference.

Due to the lack of available data and to the heterogeneity of the included studies, we could not assess how ERT affects the other outcomes defined *a priori*. From data obtained in this meta-analysis, laronidase is safe and effective for treating all phenotypes, as our included sample allows generalizability of our findings. However, studies included did not show data separately for each phenotype and some differences in response need to be further evaluated.

The inclusion criteria adopted in this systematic review did not allow the evaluation of some studies that suggested that the use of laronidase is associated to the increase in survival of patients with MPS I [29], and that this intervention, if performed at an earlier age, presents higher benefits [38–40]. Dornelles et al. [29], in a retrospective and controlled study that evaluated nine Brazilian patients without ERT and 15 patients undergoing treatment, identified that patients with MPS I in ERT presented clinically relevant lower mortality rates when compared to patients without treatment. Patients undergoing treatment had a probability of survival after seven years of onset of symptoms of 83.9%, while this rate was 41.7% for untreated patients.

In the study by Gabrielli et al. [38], two siblings diagnosed with MPS I were followed for five years. One of them started ERT with laronidase at five months (pre-symptomatic) and her sister started ERT at five years (symptomatic phase). After five years of follow-up, the brother with early onset of treatment had only corneal opacity; the sister, despite presenting clinical improvement or stabilization of some clinical manifestations, persisted with multiple dysostosis, cardiac involvement and corneal opacity. After 12 years of follow-up in ERT [39], the facial appearance, linear growth rate, and spleen and liver volumes of the sibling with onset of ERT at five months were normal. In addition, the degree of joint, vertebral and cardiac impairment were considered minimal compared to those of her sister, corroborating previous findings.

Al-Sannaa et al. [40] described a series of retrospective cases of Hurler-Scheie patients, evaluating the response to ERT in siblings with this condition. The mean age at diagnosis was 5.6 and 0.5 years for the older and younger siblings, respectively. The mean age at the beginning of ERT was 7.9 and 1.9 years for the older and younger siblings, respectively. The improvement or stabilization of signs and symptoms was more notable in younger siblings. In addition, cardiac, musculoskeletal and cognitive symptoms mostly did not develop or progress with the onset of ERT in younger siblings.

In the case of QoL, the absence of demonstrated benefit can at least in part be explained by the type of questionnaire used in some of the studies–generic questionnaires and that do not take into account specific aspects of these diseases. As far as we know, the specific QoL questionnaire for MPS is not yet validated (the MPS-HAQ).

We aimed to include an evaluation of cost-effectiveness. However, we found no study that included this outcome. Wyatt et al. [41] addressed cost of treatment in patients with MPS I and was not included in this systematic review because they reported results of retrospective data as well. In this study, there was no statistically significant association between time on ERT and either total NHS and social-care costs, hospital-care costs or non-hospital-care costs; there was a statistically significant association between time on ERT and total NHS and social-care costs, in the opposite direction to what would be expected. There are currently no adequate cost-utility studies with an appropriate time horizon and considering the health system perspective—particularly for countries with universal health systems. Other studies conducted by our group show that patients on ERT undergo fewer medical interventions, which can lead to a reduction in direct medical costs to the publicly funded health care system [37, 42].

We point out that appropriately designed studies, with a longer period of observation in various centers around the world, should be conducted to evaluate outcomes truly relevant to the course of this disease, as quality of life and survival, as also suggested by El Dib et al. [8]. The proper reporting of data is also essential.

### Conclusions

Laronidase effectively reduces urinary GAGs excretion, LVMI and hepatomegaly. Laronidase is safe in the studied population, with generally mild adverse events. Our results also suggest a beneficial effect of this drug on ROM, specifically on shoulder flexion.

## Supporting information

S1 Table PRISMA 2009 checklist. (PDF)

## Abbreviations

MPS I: Mucopolysaccharidosis type I
LSD: Lysosomal storage disease
IDUA: alpha-L-iduronidase
GAGs: glycosaminoglycans
ERT: enzyme replacement therapy
IV: intravenous
RCT: randomized clinical Trial
RE: random-effects
CI: confidence intervals
SD: standard deviations
AHI: apnea-hypopnea index
ROM: Range of motion
6MWT: 6-minute walk test
HAQ: Health Assessment Questionnaire
CHAQ: Child Health Assessment Questionnaire
